# Predicting DDI-induced pregnancy and neonatal ADRs using sparse PCA and stacking ensemble approach

**DOI:** 10.1515/jib-2024-0056

**Published:** 2025-06-10

**Authors:** Anushka Chaurasia, Deepak Kumar, Yogita

**Affiliations:** Computer Science and Engineering, 385889 National Institute of Technology Meghalaya, Shillong, India; Computer Engineering, National Institute of Technology Kurukshetra, Kurukshetra, India

**Keywords:** DDI-induced pregnancy and neonatal ADRs, sparse PCA, stacking ensemble approach

## Abstract

Predicting Drug-Drug interaction (DDI)-induced adverse drug reactions (ADRs) using computational methods is challenging due to the availability of limited data samples, data sparsity, and high dimensionality. The issue of class imbalance further increases the intricacy of prediction. Different computational techniques have been presented for predicting DDI-induced ADRs in the general population; however, the area of DDI-induced pregnancy and neonatal ADRs has been underexplored. In the present work, a sparse ensemble-based computational approach is proposed that leverages SMILES strings as features, addresses high-dimensional and sparse data using Sparse Principal Component Analysis (SPCA), mitigates class imbalance with the Multilabel Synthetic Minority Oversampling Technique (MLSMOTE), and predicts pregnancy and neonatal ADRs through a stacking ensemble model. The SPCA has been evaluated for handling sparse data and shown 2.67 %–5.45 % improvement compared to PCA. The proposed stacking ensemble model has outperformed six state-of-the-art predictors regarding micro and macro scores for True Positive Rate (*TPR*), F1 Score, False Positive Rate (*FPR*), Precision, Hamming Loss, and ROC-AUC Score with 1.16 %–14.94 %.

## 1 Introduction

Drug-Drug Interaction (DDI) denotes the interaction between two or more drugs when taken together, which can affect their effectiveness or increase the risk of adverse effects [1], [2], [3]. This has been a significant concern in clinical pharmacology, often leading to adverse drug reactions (ADRs) that have severely impacted patient health [4]. This risk has been further amplified by the fact that pregnant women often take multiple medications to manage chronic diseases, pregnancy-related complications, and other conditions, increasing the likelihood of DDIs [5]. The proportion of women prescribed two or more medications during pregnancy varies between 4.9 % and 62.4 %, with a median value of 22.5 %. The prevalence during the first trimester has varied between 4.9 % and 33.7 % [6]. Additionally, another study of 127 hypertensive pregnant women has found that 55.12 % have had potential drug-drug interactions, with 82.35 % being clinically relevant. Most of these interactions have been classified as moderate in severity (81.17 %), with 1.18 % classified as severe [5]. However, these approaches are often time-intensive and may only detect DDIs after adverse outcomes have occurred. This issue is especially critical in pregnancy, where both the mother and fetus are involved. Additionally, these approaches are constrained by the impracticality of testing all drug combinations in pregnant women due to ethical restrictions, which increases the risk of undetected DDIs [7].

Previously, many computational methodologies utilizing statistical models, machine learning, and deep learning have been proposed for predicting DDI-induced ADRs employing diverse descriptors [8], 9]. These models differ in their approach to feature representation and predictive techniques. Vilar et al. [10] explored molecular structure similarities to predict ADDIs, while Liu et al. [8] developed statistical models based on drug pair-protein interactions. Raja et al. [11] developed a robust framework integrating drug-gene interactions (DGIs) to improve the prediction of DDI-induced ADRs. The study employed multiple classifiers, including Random Forest (RF), Decision Trees, and K-Nearest Neighbors, to enhance prediction accuracy. Zhang et al. [12] integrated chemical and biological drug properties to predict DDI-induced ADRs, applying PCA to both drugs for dimensionality reduction. The predictions were made using models such as SVM, Logistic Regression (LR), KNN, and RF. Recent deep learning approaches, such as MS-ADR by Luhe et al. [13], combine multiple biomedical object views and drug-signed networks for improved prediction accuracy. Similarly, Heba et al. [14] employed biological and structural data alongside logistic regression for ADR prediction, and Zhu et al. [15] developed a deep attributed embedding method for learning drug relationships. Furthermore, Raziyeh et al. [16] utilized Jaccard similarity and neural networks to predict ADRs based on features such as side effects, enzymes, and pathways. Asfand et al. [17] introduced the Multi-Modal Convolutional Neural Network (MCNN-DDI) framework, which achieved 90 % accuracy in predicting DDI-associated events by integrating various drug features, including chemical substructures and target information. Abide et al. [18] introduced PU-GNN, a graph neural network method that uses a novel biclustering algorithm and positive-unlabeled learning to predict drug polypharmacy.

While these computational approaches have made significant strides in DDI-induced ADR prediction, most models focus on general ADRs across system organ classes, with only a few addressing pregnancy-specific ADRs [8], 19]. Despite the severe risks, predicting DDI-induced ADRs during pregnancy and in neonates remains under-explored, largely due to limited clinical data and the complexity of biological processes. In the present work, a computational approach for predicting pregnancy and neonatal ADRs considering the DDI where each drug is represented by the chemical structure in the form of SMILES (Simplified Molecular Input Line Entry System) strings. For this purpose the Sparse Principal Component Analysis (SPCA) is used to handling high-dimensional sparse data, Multilabel Synthetic Minority Oversampling Technique (MLSMOTE) has been used to address the class imbalance issue and Stacking Ensemble models is design to predict DDI-induced pregnancy and neonatal ADRS. The effectiveness of SPCA has been analyzed through dimensionality reduction and performance metrics and compared to the conventional PCA technique. Additionally, the proposed stacking ensemble model has been evaluated in comparison to six machine learning techniques, namely KNN, Extra Trees Classifier (ETC), Random Forest (RF), Decision Tree (DT), XGBoost, and CatBoost using micro and macro *TPR*, F1-Score, *FPR*, and Precision as evaluation metrics. Additionally, Hamming Loss and ROC-AUC have been employed as distinct performance indicators to further validate the model’s accuracy and effectiveness in predicting pregnancy-related ADRs.

The subsequent sections of this work are structured as follows: Section 2 delineates the problem statement pertinent to the present study. Section 3 describes the proposed methodology for predicting pregnancy and neonatal ADRs. Section 4 presents the experimental findings. Section 5 discusses the results, and finally, Section 6 concludes the study.

## 2 Problem statement

Let a set of drug pairs be represented by *DP* = {(*Fv*_1_, *ML*_1_), (*Fv*_2_, *ML*_2_), …, (*Fv*_*n*_, *ML*_*n*_)}, where each drug pair *DP*_*m*_ is described by a 2,048-dimensional feature vector *Fv*_*m*_, consisting of chemical descriptors extracted from the PubChem database [20]. Each feature vector is represented as $Fv_{m}=\left\{Fv_{m1},\right.Fv_{m2},\ldots ,Fv_{\mathrm{m2048}}$ . Each drug pair *DP*_*m*_ is associated with a multi-label vector *ML*_*m*_, representing the presence or absence of distinct pregnancy and neonatal-related ADRs. The data for these ADRs have been extracted from the TWOSIDES database [21]. Each *DP*_*m*_ is assigned a label *ML* ∈ {0, 1}^*P*^, where *P* is the total number of possible ADRs. The objective is to learn a function *f* that maps the feature vector *Fv*_*m*_ to the corresponding set of ADR labels, as shown in Eq. (1).

(1) $$f:R^{2,048}\to {\left\{0,1\right\}}^{P}$$

The dataset is defined in Eqs. (2) and (3).

(2) $$DP=\left\{\left(Fv_{m},ML_{m}\right)|m=1,2,\ldots ,n\right\}$$

(3) $$f\left(Fv_{m}\right)=ML_{m},\;\forall m=1,2,\ldots ,n$$

Here, *f* is the prediction function that maps each drug pair’s feature vector *Fv*_*i*_ to its corresponding label *ML*_*i*_, indicating the presence or absence of ADRs related to pregnancy and neonatal outcomes. The details of the chemical descriptors and the mapping of drug pairs to their ADR labels have been elaborated in Section 3.

## 3 Proposed methodology

**Figure 1:**
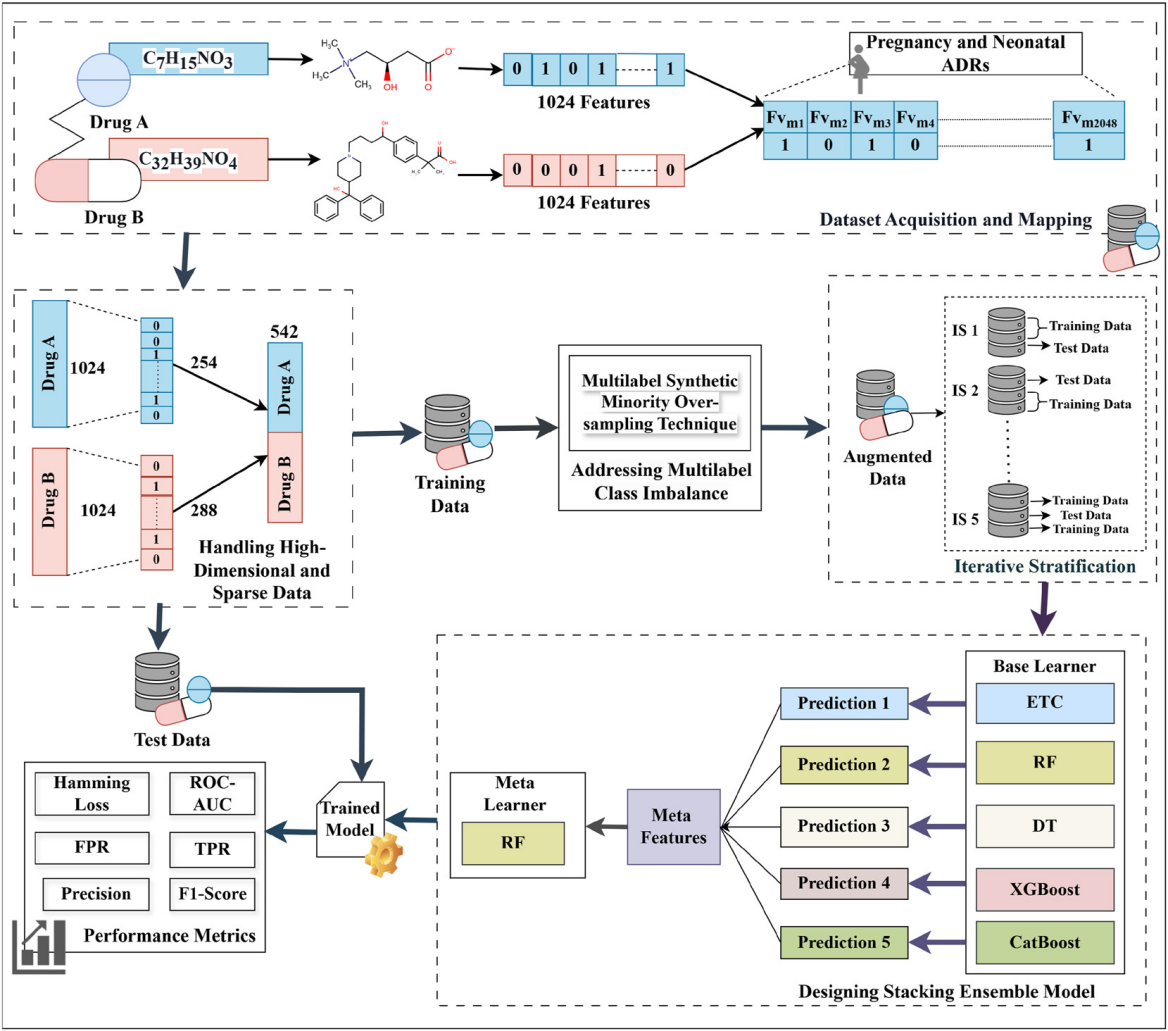
Framework of the proposed sparse ensemble-based computational approach.

The working architecture of the proposed sparse ensemble-based computational approach for predicting DDI-induced pregnancy and neonatal ADRs using chemical features is depicted in Figure 1. The approach comprises four submodules viz. Dataset Acquisition and Mapping, Handling High-Dimensional and Sparse Data, Addressing Multilabel Class Imbalance, and the Proposed Stacking Ensemble Model. Each submodule is explained in detail in the following subsections.

### 3.1 Dataset acquisition and mapping

The dataset used in this work for predicting DDI-induced pregnancy and neonatal ADRs was obtained from the TWOSIDES database, which contains 1,318 types of side effects across 63,473 drug combinations [21]. From this dataset, 17 pregnancy and neonatal ADRs, namely breast abscess, missed abortion, spontaneous abortion, cerebral palsy, eclampsia, ectopic pregnancy, failure to thrive, gestational diabetes, high-risk pregnancy, hyperglycemia, neonatal hypoglycemia, neonatal jaundice, neonatal respiratory distress syndrome, pregnancy-induced hypertension, premature separation of the placenta, retinopathy of prematurity, and stillbirth have been extracted as these ADRs are present in the dataset and are treated as a multilabel classification task. In this dataset, DDI events are represented as a four-tuple structure: (Drug A, Drug B, Total Features, ADRs). SMILES strings have been used as feature vectors, extracted from the PubChem database using the PubchemPy Python library [20]. These strings have been represented as ECFP4 fingerprints, which are 1,024-bit binary vectors where each element (0 or 1) indicates the absence or presence of a specific substructure, and have been generated using the Python-based RDKit library. The feature vectors of both drugs in each pair have been concatenated to form a combined feature vector of 2,048 dimensions (1,024 + 1,024), as shown in Table 1. This combined feature vector has been mapped to the 17 extracted pregnancy and neonatal ADRs, providing a comprehensive representation of drug-induced adverse reactions resulting from polypharmacy. Furthermore, the total number of samples and class imbalance ratio in the test dataset have been included in the Supplementary Material titled “Appendix-1”.

**Table 1:** Statistics of the dataset.

	# Features	# Samples	# After dimensionality reduction	# ADRs	Sparsity
Drug A	1,024	6,766	254	17	95.11 %
Drug B	1,024	6,766	288	17	96.11 %
Total	2,048 features		542 features		

### 3.2 Handling high-dimensional and sparse data

High-dimensional and sparse data refers to datasets that have a large number of features (high dimensionality), where a significant portion of the feature values are zero (sparsity). As illustrated in Table 1, the dataset contained a total of 2,048 binary features, with a sparsity of 95.11 %, indicating that the majority of feature values are zeros. Traditional PCA does not inherently consider sparsity and projects data onto a dense subspace, potentially losing crucial information by distributing variance across many correlated features.

The Sparse Principal Component Analysis (SPCA) technique has been employed to address this issue, introducing a sparsity constraint to ensure that each principal component depends only on a small subset of the original features [22]. The aim of this technique is to maximize the variance explained by a direction represented by the principal component vector $c\in R^{z}$ in a *z*-dimensional feature space, while limiting the number of non-zero elements in *c* as shown in Eq. (4).

(4) $$\max c^{T}\Sigma c\;\text{subject to}\;\|c{\|}_{2}=1,\;\|c{\|}_{0}\leq g$$

where the term *c* ^*T*^Σ*c* reflects the variance explained by the data in the direction of *c*, the constraint ‖*c*‖_2_ = 1 ensures that *c* is a unit vector, maintaining consistency in the principal component’s scale, while ‖*c*‖_0_ ≤ *g* promotes sparsity by limiting the number of non-zero elements in *c* to a maximum of sparsity (*g*). This balances the goal of variance maximization with dimensionality reduction, making Sparse PCA particularly suitable for high-dimensional data by focusing on a small, relevant subset of features. After applying SPCA separately to Drug A and Drug B, the most informative features were retained while preserving at least 95 % of the variance in the original feature space. As a result, SPCA selected 254 features for Drug A and 288 features for Drug B, leading to a total of 542 features per drug pair after concatenation.

### 3.3 Addressing multilabel class imbalance

**Figure 2:**
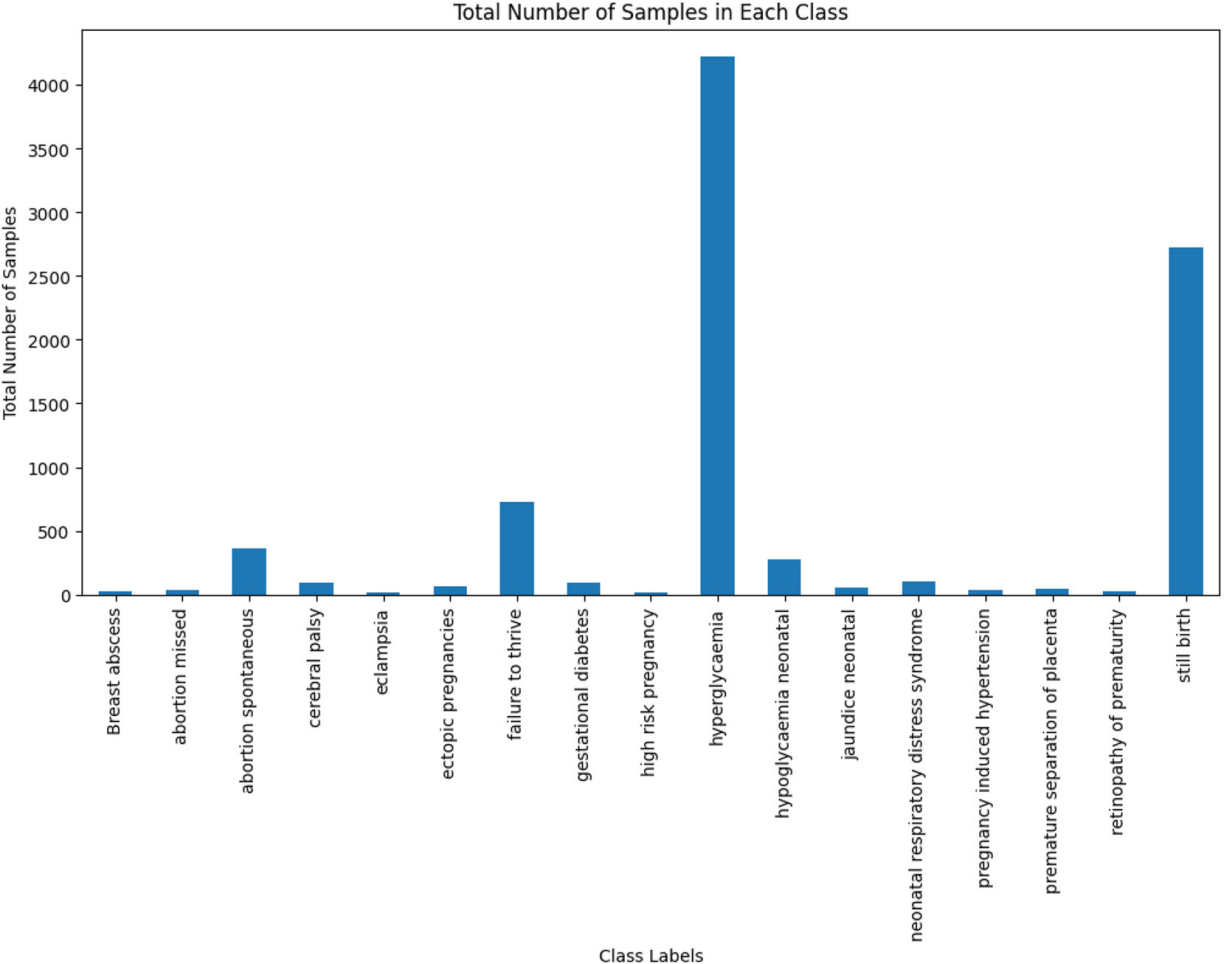
Total number of samples in each class.

The multi-label class imbalance emerges when certain labels have been significantly underrepresented relative to others, making it challenging for computational models to accurately predict minority labels as they have tended to focus on the more frequent ones. For instance, a significant class imbalance has been shown in Figure 2, with most of the samples attributed to “hyperglycaemia” and “still birth,” while “eclampsia” and “septic abortion” have been underrepresented, which leads to challenges in accurately predicting rare outcomes. To address this challenge, the Multi-label Synthetic Minority Oversampling Technique (MLSMOTE) has been employed, which calculates the imbalance ratio per label (IRLbL) for each label in the dataset, comparing it to the mean imbalance ratio to identify minority labels [23]. Once the minority labels have been isolated, the method computes the Euclidean distance between the minority instances and their nearest neighbors to select a set of ‘*k* = 5’ neighboring instances. Synthetic samples have been generated with these neighbors by interpolating the feature space while preserving the original property dimensions of the instances. These synthetic samples are labeled using a majority voting mechanism based on the labels of their neighbors, ensuring the class distribution is balanced. Finally, the newly created synthetic samples are seamlessly merged into the primary dataset and have been termed augmented data. Additionally, the total number of samples before and after applying MLSMOTE in the training dataset, and the class imbalance ratio before and after applying MLSMOTE in the training dataset have been provided in the Supplementary Material titled “Appendix-1”.

### 3.4 Designing stacking ensemble model

To predict DDI-induced pregnancy and neonatal ADRs, a stacking ensemble model has been designed. This model employed a two-level approach, where the five base models at the first level namely Decision Tree (DT), Random Forest (RF), Extra Trees Classifier (ETC), XGBoost, and CatBoost have made predictions, which have been passed to a meta-learner that is RF at the second level for final predictions. These models have been integrated into the stacking ensemble due to their robust capabilities in handling multi-label datasets. These models excel at capturing intricate label dependencies and complex feature interactions, while efficiently managing high-dimensional data. The procedure commences with the training of each base model utilizing the identical training dataset (*X*_train_, *Y*_train_), where *X*_train_ represents the features and *Y*_train_ represents the labels. Each base model (*BM*_*i*_) learns patterns in the data and generates predictions, as shown in Eq. (5).

(5) $$Pred_{i}=BM_{i}\left(X_{\text{train}}\right)$$

The predictions, denoted as *Pred*_*N*_, where *Pred*_*i*_ is the prediction of *i*-th (*BM*_*i*_) for the training data, are combined into a blended dataset. Each prediction is stacked horizontally to form *X*_blended_train_ = [*Pred*_1_, *Pred*_2_, …, *Pred*_*N*_] This blended dataset, *X*_blended_train_, has been employed to train the meta-learner (Random Forest) along with the original labels *Y*_train_ as shown in Eq. (6).

(6) $$M_{\text{meta}}=\text{RF}\left(X_{\text{blended\_train}},Y_{\text{train}}\right)$$

The *M*_meta_ learns from the combined predictions of the *BM*_*i*_ and refines these predictions by making the final prediction. During the testing phase, each *BM*_*i*_ generates predictions $Pred_{{\text{test}}_{i}}$ on the new test dataset (*X*_test_). These $Pred_{{\text{test}}_{i}}$ are similarly combined into a blended test dataset as presented in Eq. (7).

(7) $$X_{\text{blended\_test}}=\left[Pred_{{\text{test}}_{1}},Pred_{{\text{test}}_{2}},\ldots ,Pred_{{\text{test}}_{N}}\right]$$

The *M*_meta_ processes this *X*_blended_test_ dataset to make the final prediction.

## 4 Experimental setup

The experiments have been conducted on a server featuring an Intel Xeon Silver 4216 CPU (2.10 GHz), 128 GB of RAM, and HDD storage, providing the computational resources necessary for efficient training and evaluation of the models. The proposed approach has been implemented primarily using the scikit-learn library, with XGBoost and CatBoost models integrated through their respective Python packages. The dataset has been partitioned into training and test sets using an 80 %–20 % split. Stratified sampling has been applied to ensure that the distribution of ADR labels has remained consistent in both sets. This has resulted in 5,412 training samples and 1,354 test samples. For model generalization, five-fold multilabel iterative stratification has been employed, which splits the dataset while preserving label distribution across each fold. Table 2 presents the parameter configuration employed for the stacking ensemble model, highlighting the strategic decisions made to maximize its predictive capabilities.

**Table 2:** Parameter settings of the stacking ensemble model.

Predictors	Parameter settings
ETC	For ETC, the Gini Index is used as the criterion, with the best splitter applied. The minimum leaf sample is set to 1, the minimum split sample to 2, and the number of estimators to 100
RF	For RF, the Gini Index is used as the criterion with the best splitter. The minimum leaf sample is set to 1, the minimum split sample to 2, maximum depth to 25, and the number of estimators to 100
DT	For DT, the Gini Index is used as the criterion with the best splitter. The max depth value is set to 18, and the minimum sample leaf is set to 1, and the minimum sample split is set to 2
XGBoost	The hyperparameters are configured as follows: max_depth is set to 20, learning_rate to 0.1, n_estimators to 50, and booster to gbtree
CatBoost	The hyperparameters are configured as follows: iterations set to 500, learning_rate to 0.1, and depth to 6

## 5 Result analysis and discussion

The performance of the design stacking ensemble model has been evaluated using distinct metrics, namely including micro (*μ*) and macro (*α*) averages for True Positive Rate (*TPR*), F1-Score, False Positive Rate (*FPR*), and Precision. *TPR* measures the proportion of actual positives correctly identified, while precision reflects the proportion of actual positives among all predicted positives. The F1-Score balances precision and TPR, whereas *FPR* captures the proportion of actual negatives incorrectly classified as positives. Micro-averaging treats all instances equally across labels, providing an overall performance assessment, while macro-averaging calculates metrics per label and averages them, offering insights into the performance of individual labels. Additionally, ROC-AUC assesses the model’s ability to distinguish between classes at varying thresholds. Simultaneously, Hamming loss (*HL*) measures the proportion of incorrect predictions, indicating the average difference between the predicted and actual set of labels. Using both micro and macro-scores, this evaluation provides a comprehensive view of the overall and label-specific performance of the model, ensuring robustness in its predictive capabilities.

**Figure 3:**
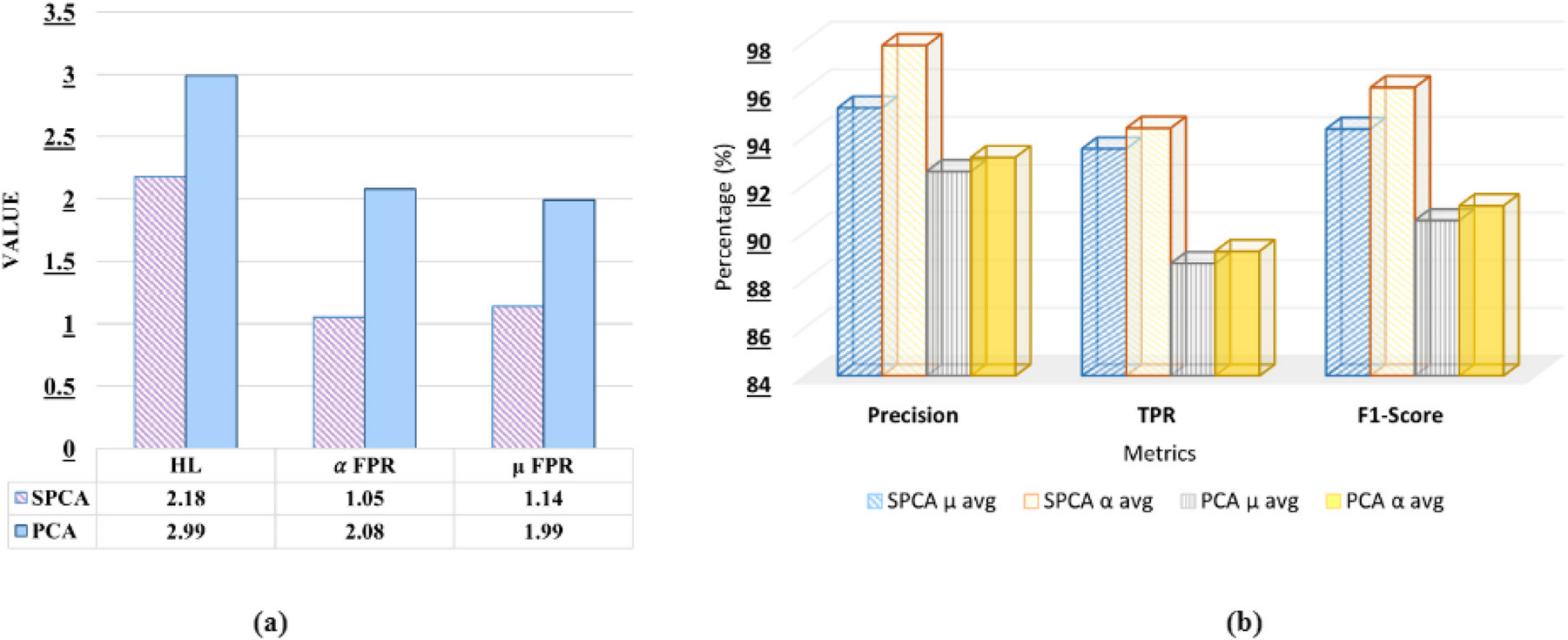
Comparative analysis of SPCA and PCA on the proposed stacking ensemble model.

The comparison of SPCA and PCA techniques for handling sparse high-dimensional data for drug pairs has been presented in Table 3. This analysis emphasizes the reconstruction error, which assesses the efficacy of the reduced data in reconstructing the original dataset, measured by Mean Squared Error sparsity after reconstruction. Furthermore, the performance comparison to predict DDI-induced pregnancy and neonatal ADR using the proposed stacking ensemble model has been shown in Figure 3, in terms of *HL*, *μ* avg and *α* avg for *FPR*, Precision, *TPR*, and F1-score.

**Table 3:** Comparison of SPCA and PCA techniques for Drug A and Drug B.

Techniques	Reconstruction error		Sparsity after reconstruction	
	Drug A	Drug B	Drug A	Drug B
SPCA	0.00306	0.00384	98.18	98.211
PCA	0.05037	0.050013	86.93	86.25

It can be observed from Table 3 that SPCA shows significantly lower reconstruction error (0.00306 and 0.00384) compared to PCA (0.05037 and 0.050013). Furthermore, SPCA maintains much higher sparsity after reconstruction (98.18 % and 98.211 %) than PCA (86.93 % and 86.25 %). These results indicate that SPCA provides a more accurate data representation and improved sparsity, making it a more effective method for dimensionality reduction.

It can be observed from Figure 3(a) that SPCA exhibits a lower *HL* value (2.18) compared to PCA (2.99), indicating better classification accuracy, as lower *HL* reflects fewer misclassifications across multiple labels. Furthermore, SPCA demonstrates lower *α* avg *FPR* and *μ* avg *FPR* compared to PCA (1.05, 1.14, 2.08, and 1.99, respectively), suggesting that SPCA generates fewer false positives both at the label level and the overall instance level. Furthermore, from Figure 3(b) it can be observed that 2.67 % higher *μ* avg precision and 4.70 % higher *α* avg precision compared to PCA. In terms of *TPR*, SPCA exceeds PCA by 5.45 % (*μ* avg) and 5.17 % (*α* avg), and for the F1-score, SPCA is higher by 4.25 % (*μ* avg) and 4.96 % (*α* avg). From the above observation, it can be concluded that SPCA consistently provides better classification performance than PCA, with gains ranging from 2.67 % to 5.45 %, depending on the metric.

**Figure 4:**
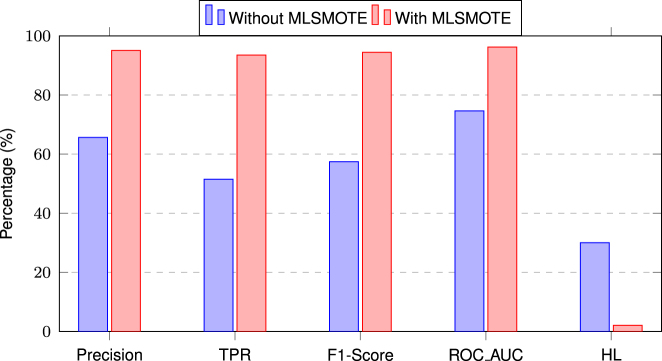
Performance comparison of the proposed stacking ensemble model with and without MLSMOTE.

It can be observed from Figure 4 that MLSMOTE has significantly improved the model’s performance. Specifically, there is an increase of 30 % in Precision, 42 % in TPR, 37 % in F1-score, and 22 % in ROC-AUC, while Hamming Loss has decreased by 28 %, indicating fewer misclassifications. These improvements highlight the impact of handling data imbalance before training a proposed model.

**Figure 5:**
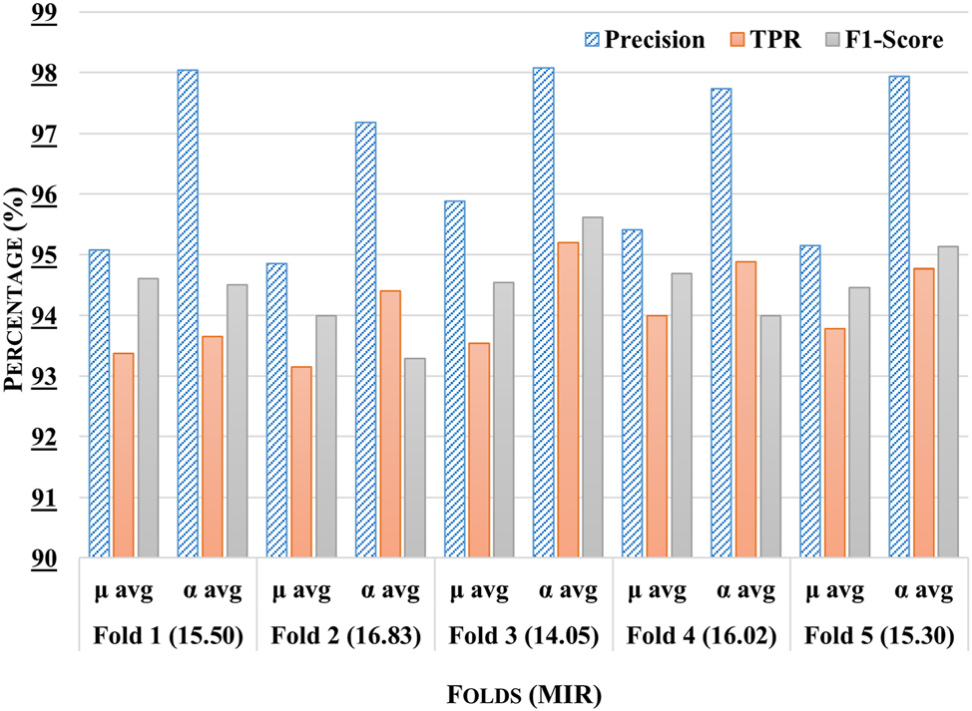
Comparative analysis of the proposed stacking ensemble model across different folds with mean imbalance class ratio (MIR) for each fold.

The comparative analysis of the proposed stacking ensemble model across five distinct experimental subsets has been shown in Figure 5. It has been observed that fold 3 has exhibited superior performance across all metrics, with a *μ* avg precision of 97.5 %, a *TPR* of 96.75 %, and an F1-Score of 96.1 %, demonstrating the model’s robust predictive capability for this subset. Furthermore, the precision of *α* avg has surpassed 97 %, further highlighting the model’s effectiveness on different labels. In contrast, fold 2 has demonstrated slightly lower performance, with a *μ* avg precision of 94.62 % and a *TPR* of 92.36 %, suggesting that although the model is still effective, it does not reach the peak performance seen in fold 3. It has been observed that Fold 3 has demonstrated the highest classification performance, surpassing all other folds, which has been attributed to its lowest mean class imbalance ratio (14.05). This improved balance has enabled the model to learn minority class patterns more effectively, reducing bias toward majority classes. In contrast, Folds 2 and 5 have exhibited higher imbalance ratios (16.83 and 15.30, respectively), likely impacting their classification performance.

**Figure 6:**
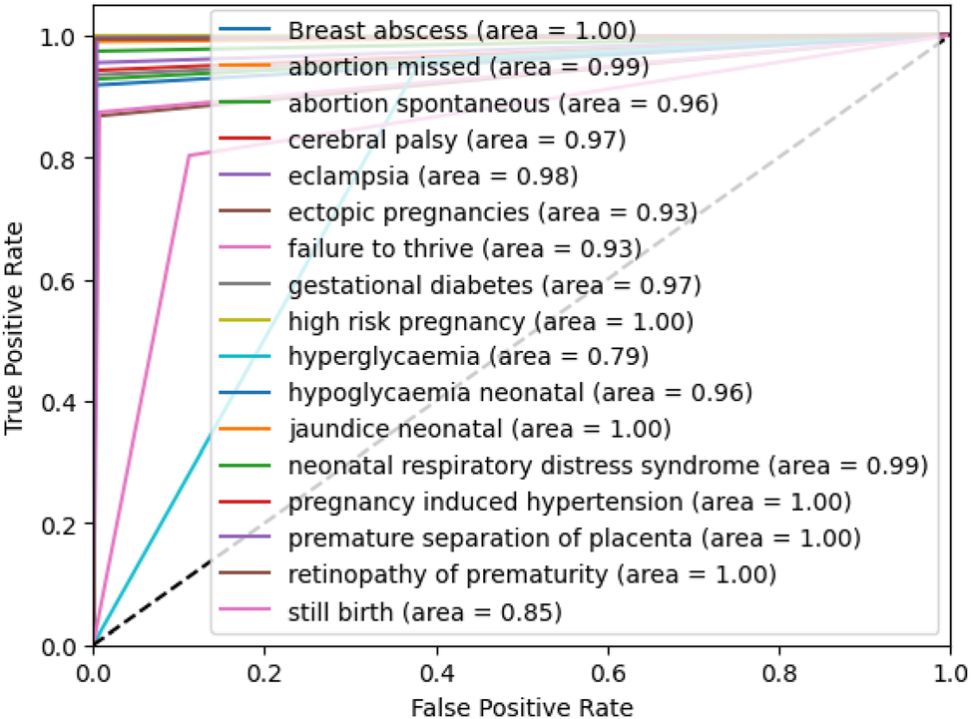
ROC-AUC of the proposed stacking ensemble model on third fold.

The ROC-AUC curve of all ADRs for the proposed stacking ensemble model within the third fold has been shown in Figure 6. The ROC curve illustrates the performance of the model in predicting various pregnancy-related and neonatal conditions. Several conditions, such as breast abscess, high-risk pregnancy, neonatal jaundice, pregnancy-induced hypertension, prematurity retinopathy, and premature separation of the placenta, have achieved a perfect AUC of 1.00, indicating excellent prediction precision. Conditions such as abortion missed, and neonatal respiratory distress syndrome have also performed very well, with AUCs of 0.99. However, conditions such as stillbirth and hyperglycemia have shown lower AUCs of 0.85 and 0.79, suggesting that the model has struggled more with these cases. It can be concluded that the model has shown strong performance, ranging from 0.93 to 1.00, across all labels except for two.

The proposed stacking ensemble model has been compared with six state-of-the-art methods, namely KNN, ETC, RF, DT, XGBoost, and CatBoost, in terms of Precision, *TPR*, *FPR*, and F1-Score, using both *μ* and *α* avg as illustrated in Table 4. It is observed that the proposed model has consistently outperformed the other methods, achieving the highest *μ* avg precision of 95.23 %, exceeding the next best model (RF at 89.60 %) by 5.63 %. Similarly, the *μ* avg precision of 97.85 % surpasses RF by 1.16 %. For *TPR* the proposed model has reached 93.51 % (*μ* avg) outperforming CatBoost by 2.20 %, while a *α* avg *TPR* of 94.38 % exceeds CatBoost by 4.29 %. The lowest *μ* avg *FPR* of 1.32 % has been achieved, improving over RF by 0.54 %, and the *α* avg *FPR* of 2.18 % is 1.81 % lower than CatBoost. In terms of F1-Score, the model has attained a *μ* avg score of 94.46 %, 4.83 % higher than CatBoost, and a macro-average score of 94.64 %, surpassing RF by 8.34 %. Conversely, KNN shows the weakest performance, with the lowest *μ* avg precision of 80.29 %, which is 14.94 % less than the Proposed Model, and the highest *α* avg *FPR* of 5.22 %, which is 3.04 % worse than the Proposed Model. It can be stated that the Proposed Stacking Ensemble Model demonstrates superior performance across all metrics, with the best balance of high precision and low false positives, making it more reliable than other models in this comparison.

**Table 4:** Performance comparison of the proposed ensemble model with state-of-the-art methods (±SD).

Models	Metrics	Precision (±SD)	TPR (±SD)	FPR (±SD)	F1-score (±SD)
KNN	*μ* avg	80.29 ± 0.0118	82.84 ± 0.0061	3.049 ± 0.0020	84.06 ± 0.0073
	*α* avg	89.25 ± 0.0138	88.99 ± 0.0031	5.22 ± 0.0033	84.57 ± 0.0015
ETC	*μ* avg	89.09 + 0.0043	83.40 + 0.0047	1.98 + 0.0007	86.11 + 0.0030
	*α* avg	95.11 + 0.0038	85.73 + 0.0047	4.06 + 0.0016	86.30 + 0.0078
RF	*μ* avg	89.60 ± 0.0093	83.26 + 0.0075	1.86 + 0.0013	86.11 + 0.0032
	*α* avg	96.69 ± 0.0040	84.64 + 0.0122	4.09 + 0.0015	86.30 + 0.0078
DT	*μ* avg	87.32 ± 0.0068	89.45 ± 0.0021	2.12 + 0.0122	91.53 ± 0.0043
	*α* avg	90.22 ± 0.0168	85.39 ± 0.0218	3.99 ± 0.0011	87.23 ± 0.0102
XGBoost	*μ* avg	88.88 + 0.0041	84.79 + 0.0057	2.05 + 0.0008	89.45 ± 0.0214
	*α* avg	95.41 + 0.0045	87.17 + 0.0035	4.44 + 0.0021	86.77 + 0.0046
CatBoost	*μ* avg	89.06 ± 0.0067	91.31 ± 0.0044	3.42 ± 0.0032	89.63 ± 0.0139
	*α* avg	90.23 ± 0.0032	90.09 ± 0.141	3.99 ± 0.0253	88.91 ± 0.0145
Proposed model	*μ* avg	95.23 ± 0.0044	93.51 ± 0.0036	1.32 ± 0.0013	94.46 ± 0.0027
	*α* avg	97.85 ± 0.0043	94.38 ± 0.0049	2.18 ± 0.0022	94.64 ± 0.0036

*μ* avg.: micro average, *α* avg.: macro average.

Table 5 has presented an ablation study evaluating the performance of the proposed stacking ensemble model using different meta-learners. The evaluation has been based on three key metrics: F1-score, ROC-AUC, and HL. The results have indicated that RF has achieved the highest performance, with an F1-score of 94.64 %, ROC-AUC of 96.23 %, and the lowest HL of 2.08. ETC and CatBoost have also demonstrated competitive performance, with an F1-score of 90.30 % and 86.01 %, respectively. In contrast, XGBoost has exhibited the lowest performance, with an F1-score of 81.91 % and ROC-AUC of 77.20 %. The findings have highlighted the importance of selecting an appropriate meta-learner, with RF emerging as the most effective choice for improving classification performance in the stacking ensemble framework.

**Table 5:** Ablation study of the proposed stacking ensemble model using different meta-learners.

Meta learners	F1-score	ROC-AUC	HL
DT	85.53 ± 0.0012	88.06 ± 0.0047	3.29 ± 0.0011
ETC	90.30 ± 0.009	86.52 ± 0.0060	4.26 ± 0.0016
CatBoost	86.01 ± 0.0096	89.21 ± 0.0042	3.05 ± 0.0253
XGBoost	81.91 ± 0.0005	77.20 ± 0.0146	3.99 ± 0.0121
RF	94.64 ± 0.0036	96.23 ± 0.0016	2.08 ± 0.0023

## 6 Conclusions

In the present work, a sparse PCA and stacking ensemble-based approach has been proposed for analyzing the DDI-induced pregnancy and neonatal ADR prediction capabilities of chemical drug properties. In comparison to conventional PCA, the sparse PCA method produced enhanced outcomes, improving dimensionality reduction and increasing performance by 2.67 %–5.45 %. Furthermore, the stacking ensemble model exhibited remarkable performance when benchmarked against six state-of-the-art predictors, with improvements ranging from 1.16 % to 14.94 %, underscoring its potential for more accurate predictions. Future research will incorporate distinct drug properties for better feature representation, integrate advanced deep learning for improved interaction learning, and enhance model transparency through interpretable AI to provide clinically relevant insights into DDI-induced pregnancy-related ADRs. This expansion will enhance the robustness and predictive accuracy of the model in real-world scenarios.

## Supplementary Material

Supplementary Material Details
