# Supplementary material for: Predicting DDI-induced pregnancy and neonatal ADRs using sparse PCA and stacking ensemble approach
Source: J Integr Bioinform. 2025 Jun 10;22(2):20240056. doi: 10.1515/jib-2024-0056 (PMC12569586; doi:10.1515/jib-2024-0056)
Supplement: Supplementary file 1 — Supplementary Material Details [file j_jib-2024-0056_suppl_001.pdf]

Predicting DDI-Induced Pregnancy and Neonatal ADRs Using Sparse PCA and Stacking Ensemble Approach

Anushka Chaurasia[1]*, Deepak Kumar[1], Yogita[2]

[1] Computer Science and Engineering, National Institute of Technology Meghalaya, India

[2] Computer Engineering, National Institute of Technology Kurukshetra, India

Table 1: Total No. of samples Before and After Applying MLSMOTE in Training Dataset

S.No	ADRs	Total No. of samples Before MLSMOTE	Total No. of samples After MLSMOTE
1	Breast abscess	24	887
2	Abortion missed	29	1125
3	Abortion spontaneous	292	1900
4	Cerebral palsy	74	395
5	Eclampsia	10	99
6	Ectopic pregnancies	57	987
7	Failure to thrive	605	2332
8	Gestational diabetes	77	736
9	High risk pregnancy	15	549
10	Hyperglycaemia	3395	6783
11	Hypoglycaemia neonatal	225	1294
12	Jaundice neonatal	36	1763
13	Neonatal respiratory distress syndrome	84	1231
14	Pregnancy induced hypertension	30	1725
15	Premature separation of placenta	40	1643
16	Retinopathy of prematurity	17	537
17	Still birth	2175	4734
**Total no. of samples**		**5412**	**18030**

Table 2: Total No. of Samples and Class Imbalance Ratio On Test Data.

S.No	ADRs	Total No. of Samples	Class imbalance Ratio
1	Breast abscess	4	208
2	Abortion missed	5	166.4
3	Abortion spontaneous	72	11.55556
4	Cerebral palsy	19	43.78947
5	Eclampsia	3	277.3333
6	Ectopic pregnancies	6	138.6667
7	Failure to thrive	124	6.709677
8	Gestational diabetes	17	48.94118
9	High risk pregnancy	3	277.3333
10	Hyperglycaemia	832	1
11	Hypoglycaemia neonatal	54	15.40741
12	Jaundice neonatal	15	55.46667
13	Neonatal respiratory distress syndrome	22	37.81818
14	Pregnancy induced hypertension	5	166.4
15	Premature separation of placenta	8	104
16	Retinopathy of prematurity	4	208
17	Still birth	547	1.521024

Table 3: Class Imbalance Ratio before and After Applying MLSMOTE on Training Dataset

S.No	ADRs	Class imbalance Ratio Before MLSMOTE	Class imbalance Ratio After MLSMOTE
1	Breast abscess	240.60	10.49
2	Abortion missed	197.97	8.064
3	Abortion spontaneous	17.58	4.36
4	Cerebral palsy	71.74	24.81
5	Eclampsia	519.38	102.0
6	Ectopic pregnancies	106.38	33.33
7	Failure to thrive	8.27	3.37
8	Gestational diabetes	70.96	12.85
9	High risk pregnancy	374.83	17.57
10	Hyperglycaemia	1.66	1.98
11	Hypoglycaemia neonatal	23.24	6.88
12	Jaundice neonatal	131.64	4.78
13	Neonatal respiratory distress syndrome	62.82	7.28
14	Pregnancy induced hypertension	192.28	4.91
15	Premature separation of placenta	139.93	5.20
16	Retinopathy of prematurity	321.14	17.98
17	Still birth	1.48	1.15
**Mean Class Imbalance Ratio**		**145.99**	**15.71**
